# Parasite diversity of European *Myotis* species with special emphasis on *Myotis myotis* (Microchiroptera, Vespertilionidae) from a typical nursery roost

**DOI:** 10.1186/s13071-015-0707-7

**Published:** 2015-02-15

**Authors:** Raphael Frank, Thomas Kuhn, Antje Werblow, Andrew Liston, Judith Kochmann, Sven Klimpel

**Affiliations:** Goethe-University (GU), Institute for Ecology, Evolution and Diversity; Senckenberg Biodiversity and Climate Research Centre (SBiK-F), Senckenberg Gesellschaft für Naturforschung (SGN), Max-von-Laue-Str. 13, Frankfurt am Main, D-60438 Germany; Senckenberg German Entomological Institute (SDEI), Eberswalder Str. 90, Muencheberg, D-15374 Germany

**Keywords:** *Myotis myotis*, *Spinturnix myoti*, Ectoparasites, Roosting place

## Abstract

**Background:**

Bats belong to one of the most species-rich orders within the Mammalia. They show a worldwide distribution, a high degree of ecological diversification as well as a high diversity of associated parasites and pathogens. Despite their prominent and unique role, the knowledge of their parasite-host-relationships as well as the mechanisms of co-evolutionary processes are, partly due to strict conservation regulations, scarce.

**Methods:**

Juvenile specimens of the greater mouse-eared bat (*Myotis myotis*) from a roosting colony in Gladenbach (Hesse, Germany) were examined for their metazoan endo-and ectoparasite infections and pathogens. Morphometric data were recorded and the individuals were checked for Lyssavirus-specific antigen using a direct immunofluorescence test. For unambiguous species identification, the bats were analysed by cyt-b sequence comparison.

**Results:**

*Myotis myotis* were parasitized by the six insect and arachnid ectoparasite species, i.e. *Ixodes ricinus*, *Ischnopsyllus octactenus*, *Ichoronyssus scutatus*, *Steatonyssus periblepharus*, *Spinturnix myoti* and *Cimex dissimilis*. Additionally, the nematode *Molinostrongylus alatus* and the cestode *Vampirolepis balsaci* were recorded. Each bat was parasitized by at least four species. The parasites showed partially extreme rates of infection, never recorded before, with more than 1,440 parasites per single host. *Ichoronyssus scutatus*, *Steatonyssus periblepharus*, *Vampirolepis balsaci* and *Molinostrongylus alatus* are recorded for the first time in Germany. A checklist for Europe is presented containing records of 98 parasite species of 14 *Myotis* species.

**Conclusions:**

The *Myotis myotis* from Gladenbach (Hesse, Germany) were parasitized by a diverse parasite fauna with high infestation rates. We assume that in juvenile *Myotis* the number of parasites is generally higher than in adults due to only later acquired immune competence and behavioural adaptations. Our results revealed new insights into parasite fauna of *M. myotis* and European bats in general. The finding of endoparasitic cyclophyllidean cestodes that have a two-host lifecycle is, considering the stationary behaviour of the juvenile bats, rather unusual and suggests a non-predatory transmission mechanism (e.g. via autoinfection).

A new insight gained from the collated literature was that the European wide composition of the *Myotis* parasite fauna is dominated by a few specific taxonomic groups in Europe.

## Background

As the second largest order worldwide within the Mammalia [1] bats show a high degree of ecological diversification. This variability is enabled by morphological, behavioural and physiological adaptations [2]. Paradoxically, one third of the indigenous mammalian species of Europe are bats of the suborder Microchiroptera, but details about the exact number of species or their distribution are still scarce [3]. The most diverse family is the Vespertilionidae, within which the genus *Myotis* comprises between 92 [4] and 100 species [5]. Most members of *Myotis* are distributed in the northern hemisphere [4] and at least 14 species are known in Europe. Based on genetic studies, speciation within the genus *Myotis* took place in geographically isolated populations [6]. As a result of habitat loss, several bat species populations increasingly use urbanised areas as alternative habitats [7]. The most common synanthropic species in Europe are *Myotis daubentoni*, *M. dasycneme* and *M. myotis* [8]. They live in close contact with humans and can act as vectors for several zoonotic pathogens. Several virus species, partly human pathogenic, are recorded in European bats such as coronaviruses (CoVs), filoviruses, henipaviruses and astroviruses (RNA viruses) as well as herpes-and adenoviruses (DNA viruses) [9]. The virus strains *Lyssavirus* genotype 5 and 6 (EBLV 1 and 2) (RNA-virus, Rhabdoviridae) are causing human rabies [3,8,10]. Long-term studies show that *Eptesicus serotinus* is the most common species in Germany with *Lyssavirus* infections [10], while only occasional reports of infection of *M. myotis* are known.

Beside the aforementioned virus diversity, *M. myotis* also shows a high diversity of metazoan endo-and ectoparasites. All bats in Germany are strictly protected, which severely limits opportunities for research on their parasite fauna and the number of studies in this field are comparatively low [11]. The few existing records of parasites in bats are from Eastern Europe, Austria, Switzerland, Great Britain and Spain (e.g. [12,13]) as well as Germany [14]. Because of its wide range of distribution, the parasite diversity research of *M. myotis* is of special interest in relation to the remaining 38 European bat species and is useful for other bat species to understand parasite-host ecology [10]. Another point is that ectoparasites may be capable of acting as vectors of the mentioned viruses as well as be human pathogenic. As for all other wild animals several ecological relationships are directly or indirectly linked to parasites. For instance, parasite induced avoidance strategies were observed in *M. myotis* [15]. In addition, physiological adaptations within the parasites were also postulated [16]. Beside strictly host specific parasites, several species occur on more than one *Myotis* species as well as members of other bat families [17]. Consequently, the potential parasite fauna of the European *Myotis* species is expected to be large; however, a comprehensive documentation of all known European *Myotis* species and associated parasites is missing.

The first aim of our study was to identify the parasite species of juvenile *M. myotis* from a Hessian (German) population. Our second aim was to provide a complete overview of the parasite fauna of European *Myotis.* For this*,* we compiled a parasite checklist that includes all new and previous metazoan parasite records of the 14 *Myotis* species in Europe.

## Methods

### Sampling site and collection of parasites

The study was carried out using 30 juvenile specimens of *Myotis myotis* (half males and females) which had died of natural causes. They were collected by a member of ChiroTEC [18] on July 21 in 2012 at the Martinskirche in Gladenbach, Hesse (Germany). The specimens were dead less than 48 hours. The nursery roost is located at the church’s roof truss and was monitored by members of ChiroTEC. The coordinates of the sampling site are N 50.768213, E 8.583106. In 2011, the colony consisted of about 767 estimated adult females [19].

Sex and the following main morphometric data of each bat were recorded (Table 1): total weight, head-body length, forearm and upper arm length. In addition, the weight of the heart, lung, left and right kidney, spleen, liver and filled digestive track was recorded. Bats were checked for ectoparasites first. These were separated into systematic classes and stored in 70% ethanol. A few fleas of both sexes were left in potassium hydroxide (10%) for 2 hours at 95°C or 24 hours at 35°C. Afterwards they were rinsed in xylol and stored in 70% ethanol. Bats were then dissected and checked for endoparasites. Endoparasites used for morphological species identification were placed in Histofix overnight and stored in 70% ethanol. Parasites used for molecular species identification were preserved in absolute ethanol.

**Table 1:** **Morphometrical data of** ***Myotis myotis*** **(n=30) from Gladenbach (Hesse)**

	**Total weight [g]**	**Head-Body length [cm]**		**Upper arm length [cm]**		**Forearm length [cm]**	
Min.	7.310	4.9		2.00		4.00	
Max.	14.239	6.8		3.10		5.80	
Ø	11.184 (±1.517)	6.09 (±0.429)		2.56 (±0.28)		4.93 (±0.45)	
	**Heart [g]**	**Lung [g]**	**Kidney left [g]**	**Kidney right [g]**	**Spleen [g]**	**Liver [g]**	*Digestive track [g]
Min.	0.082	0.121	0.031	0.050	0.008	0.226	0.247
Max.	0.217	0.286	0.085	0.096	0.099	0.595	0.932
Ø	0.138 (±0.027)	0.201 (±0.041)	0.067 (±0.011)	0.072 (±0.011)	0.053 (±0.020)	0.400 (±0.094)	0.551 (±0.091)

Ø=average, *filled, ± standard deviation.

### Analysis of parasite diversity

Species were identified and the quantitative parasitological data prevalence (P %), abundance (A), intensity (I), mean intensity (mI) and index of relative frequency (pi) were calculated following Bush et al. [20] (Table 2). Intensity of infection (I) is the number of individuals of a particular parasite species in a single infected host (expressed as a numerical range), whereas mean intensity of infection (mI) is the average intensity. Mean abundance (mA) is the total number of individuals of a particular parasite species in a sample of a particular host species divided by the total number of hosts of that species examined, including both infected and uninfected hosts [20]. The diversity of the metazoan parasite fauna was estimated using the Shannon-Weaver diversity index (Hs) and the evenness index (E) following Shannon & Weaver [21]. Spearman’s Rank test was used in order to analyze the relationship between the Index of Condition, body weight divided by forearm length (after Lourenco & Palmeirim [15]), and the parasite intensity (number of parasites per host specimen) with ectoparasites and cestodes, respectively, using Graphpad Prism software version 5.01. During necropsy, muscle tissue samples for species identification and brain tissue samples for *Lyssavirus* identification were taken. The parasite list was compiled in consideration of all previously published literature.

**Table 2:** **Parasitological data of** ***Myotis myotis*** **(n=30) from Gladenbach (Hesse)**

**Species**	**P [%]**	**A**	**mI**	**I**	**pi**	**St**	**Hs**	**E**
Cestoda							0.854	0.41
*Vampirolepis balsaci*	70	4.1	5.8	1-18	1.212	a		
Nematoda								
*Molinostrongylus alatus*	3.3	0.03	1	1	0.009	a		
Arachnida								
*Ichoronyssus scutatus*	100	72	72	5-165	21.126	a,l		
*Steatonyssus periblepharus*	100	228.6	228.6	26-1288	67.077	a,l		
*Spinturnix myoti*	100	8.1	8.1	1-17	2.390	a,n		
*Ixodes ricinus*	3.3	0.03	1	1	0.009	l		
Insecta								
*Cimex dissimilis*	83.3	2.8	3.3	1-17	0.821	a,l		
*Ischnopsyllus octactenus*	13.3	0.13	1	1	0.039	a		

*Abbr.: A* Abundance, *a* adult, *E* evenness index, *Hs* Shannon=Weaver diversity index [21], *I* Intensity, *l* larval, *mI* mean Intensity, *n* nymphal, *P* prevalence, pi=index of relative frequency [20], *St* developmental stage.

### Species identification

Genomic DNA was isolated and purified from small amounts of muscular tissue (10–20 mg) using an AcroPrep PALL 96-well glass fiber plate (1 ml; 1 μm) according to the instructions provided by Ivanova et al. [22]. The cytochrome b genetic marker from *Myotis* sp. was amplified using primer Cyt-b FWN (5’-TGA-TGR-AAC-TTY-GGY-TCY-CTY-YTA-GGA-RTY-T-3’) and Cyt-b REV (5’-CCR-ATR-ATR-ATR-TAK-GGR-TRY-TCD-ACD-GGT-TG-3’). PCR-reaction (50 μl) included 25 μl Master-Mix (Peqlab Biotechnology GmbH, Erlangen, Germany) containing dNTP, MgCl_2_, buffer and Taq polymerase, 3 μl of each primer (10 pmol μl^−1^), 14 μl ddH_2_O and 5 μl genomic DNA. Each PCR reaction was performed in a thermocycler (Eppendorf, Germany) under the following conditions: one cycle of initial denaturation at 94°C for 120 sec, followed by 39 cycles of 94°C, 60 sec (denaturation), 55°C, 60 sec (annealing) and 72°C for 75 sec (extension). The final extension was carried out at 72°C for 5 min. PCR products were examined on 1% agarose gels including a low range ladder marker (peqGOLD, Erlangen, Germany) to estimate the size of the PCR products. Successfully amplified PCR products were purified using the peqGOLD Cycle-Pure Kit (Peqlab Biotechnology GmbH, Erlangen, Germany) following the instructions of the manufacturer. The purified products were sequenced by Seqlab (Goettingen GmbH, Germany) using primer Cyt-b FWN (5’-TGA-TGR-AAC-TTY-GGY-TCY-CTY-YTA-GGA-RTY-T-3’). For species identification, obtained sequence data were compared with previously published Genbank data using the BLASTn algorithm [23]. Morphological parasite identification was carried out using the descriptions e.g. by Genov et al. [24], Brinck-Lindroth & Smit [25], Tian & Jin [26], Pocora et al. [27].

### Lyssavirus identification

Brain tissue of the 30 juvenile *M. myotis* and eight additional juvenile specimens from the same collecting point were checked for *Lyssavirus* infection using a direct immunofluorescence test (IFT/FAT). The eight additional specimens were in the same visible condition and age, and were also collected on July 21, 2012. These included half males and half females. Tests were carried out following the official guidelines of the Federal Research Institute for Animal Health, Friedrich-Loeffler-Institute [28].

### Review methodology

To compile a parasite-host list as complete as possible, we searched PubMed, Medline, SciELO and Google Scholar for publications that contain records of *Myotis* species and associated parasite species as well as locality of the record from Europe. Foremost, we used primary literature. If description was unambiguous and met our requirements, we also included records mentioned in reviews and other secondary literature.

## Results

### Morphometric data and species identification of *Myotis myotis*

The sympatric sibling species *Myotis myotis* and *M. blythii* are difficult to distinguish based on morphological characters, especially as juveniles. They can roost together [29]. Therefore, cyt-b segment analysis was used. In total, 27 *Myotis* specimens could be sequenced successfully and Blast-analyses revealed 99-100% identity with a sequence of *M. myotis* from Romania (Acc.: GU817367.1), suggesting that our specimens belong to the same species. We identified 27 specimens as *M. myotis* which were used for parasitological analysis. The remaining three specimens did not provide enough DNA for sequencing but were assumed to be the same species. The obtained sequences of *M. myotis* were deposited in Genbank under the accession numbers KJ765363-KJ765389. Morphometric data of the examined *Myotis* specimens are summarized in Table 1. Head-body length, fore arm length, upper arm length and total weight of bats were normally distributed (D’Agostino & Pearson normality test; p=0.181; 0.839; 0.720; 0.840). Because the colony was checked frequently and bats were collected within 48 hours, the state of preservation was good. The bats showed nearly no visible signs of decay, e.g. dried eyes or mucosa. We have secured the condition based on the findings of well-developed muscle and fat tissue we observed during section.

### Parasites of *Myotis myotis* from Gladenbach (Hesse)

**Figure 1:**
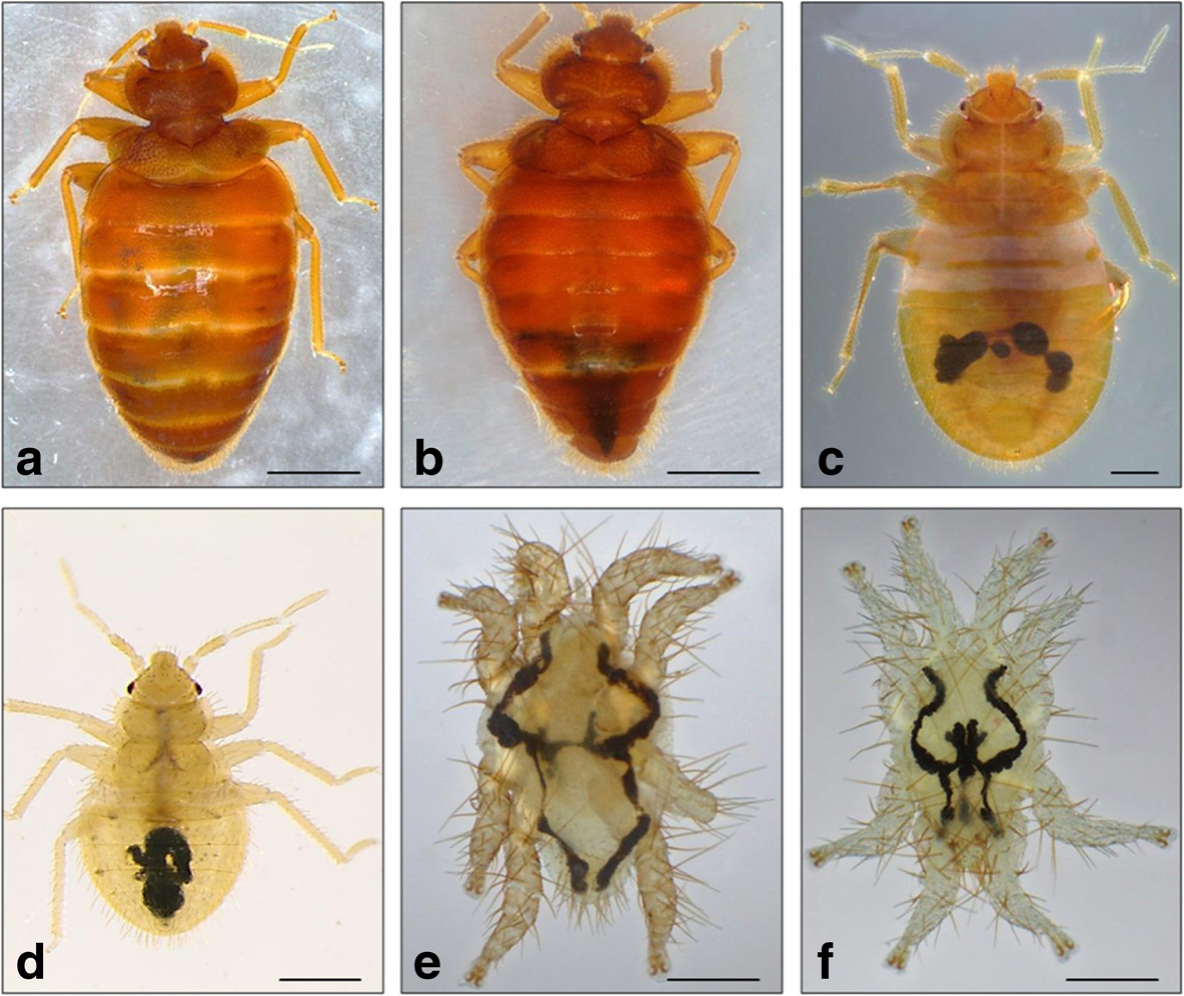
**Bugs and spinturnicid mites of** ***Myotis myotis*** **from Gladenbach (Hesse). a**-**d** *Cimex dissimilis* (Heteroptera, bugs), **e**-**f** *Spinturnix myoti* (Mesostigmata, mites). Habitus, dorsal view. Light micrographs. **a)** female, adult **b)** male, adult **c)** older larval stage **d)** younger larval stage. In bugs ingested blood (dark spots) in the digestive track is well visible in both larval stages, but hardly visible in adult stages. Scale bars: **a**-**b**=1 mm, **c**-**d**=500 μm, **e**-**f**=200 μm.

**Figure 2:**
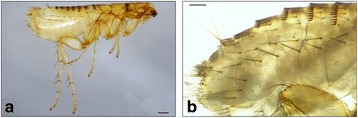
**Fleas of** ***Myotis myotis*** **from Gladenbach (Hesse).** *Ischnopsyllus octactenus* (Siphonaptera, fleas). Lateral view. Light micrographs. **a)** male, habitus, **b)** female, details of abdomen. Scale bars: **a**=200 μm, **b**=50 μm.

**Figure 3:**
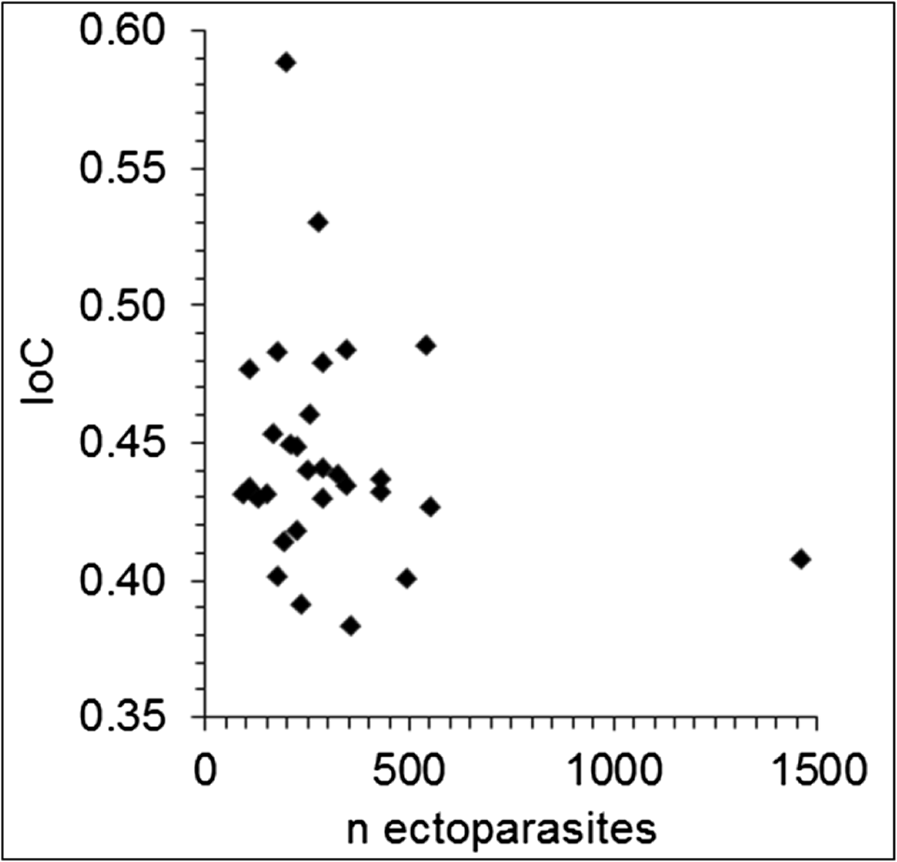
**Relationship between ectoparasite loads and the index of condition of hosts.** A correlation could not be observed. A single host was parasitized by more than 1,462 parasites. IoC=Index of Condition (body weight divided by forearm length).

**Figure 4:**
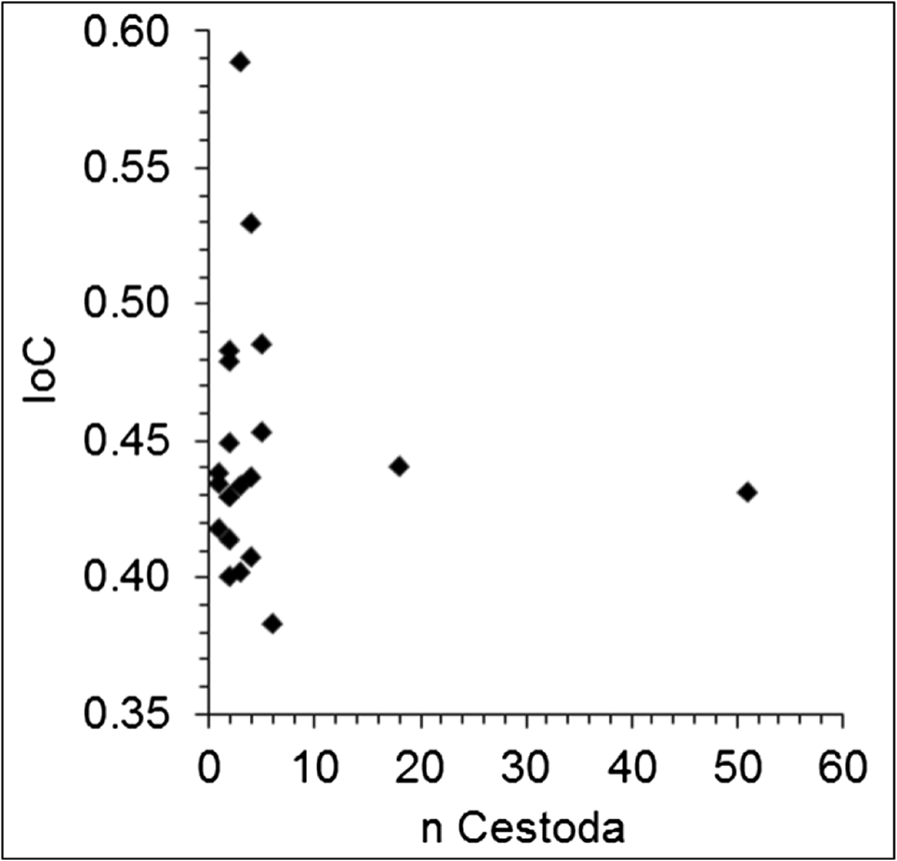
**Relationship between cestode loads and the index of condition of hosts.** A correlation could not be observed. Two hosts were parasitized by 51 and 18 *Vampirolepis balsaci* (cestode), respectively. IoC=Index of Condition (body weight divided by forearm length).

All bats in the sample were parasitized. Eight parasite species *Ixodes ricinus*, *Ischnopsyllus octactenus*, *Ichoronyssus scutatus*, *Steatonyssus periblepharus*, *Spinturnix myoti*, *Cimex dissimilis*, *Molinostrongylus alatus* and *Vampirolepis balsaci* were recorded from these *M. myotis* (Table 2). We were able to record *S. periblepharus I. scutatus*, *V. balsaci* and *M. alatus* as new records from *M. myotis* in Germany. All specimens were infected with the mite species *I. scutatus*, *S. myoti* (Figure 1) and *S. periblepharus*. Infection parameters of *C. dissimilis* (Figure 1) and *V. balsaci* reached a prevalence of 83.3% and 70%, respectively. We were also able to find *I. octactenus* (Figure 2) on 13.3% of the individuals. *Ixodes ricinus* and *M. alatus* each showed a prevalence of 3.3%. Statistical analyses revealed no significant correlation between the index of condition [15] and the total ectoparasite as well as cestode parasite load of the bats (Spearman, non-parametric test: P=0.56, P=0.68; α=0.05) (Figures 3 and 4). No sex-based differences in parasitism patterns (total numbers of ecto-/endoparasites) were observed (two-tailed, Mann–Whitney U-test; P=0.22; α=0.05).

### Virus detection

The brain tissue of the 30 juvenile greater mouse-eared bats and additional 8 specimens from the same roosting place showed no positive results by using the direct immunofluorescence test (IFT). There was no evidence of *Lyssavirus*-specific antigen.

### Parasites of *Myotis* in Europe

**Figure 5:**
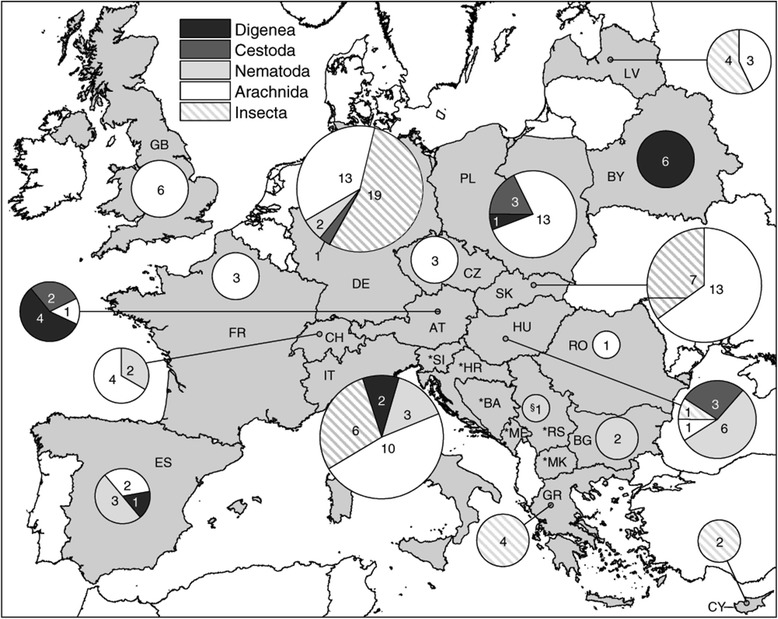
**Country-specific composition of European parasite fauna of** ***Myotis.*** The numbers within the circles show the number of *Myotis* parasite species. Size of the circles depends on the total number of the country specific parasite fauna in relation to the total number of *Myotis* parasites from Europe (n=98). Records are given in Table 3. Abbreviations: AT=Austria, *BA=Bosnia and Herzegowina, BG=Bulgaria, BY=Belarus, CH=Switzerland, CY=Cyprus, CZ=Czech Republic, DE=Germany, ES=Spain, FR=France, GB=Great Britain, GR=Greece, *HR=Croatia, HU=Hungary, IT=Italy, LV=Latvia, *ME=Montenegro, *MK=The former Yugoslav republic of Macedonia, PL=Poland, RO=Romania, *RS=Serbia, *SI=Slovenia, SK=Slovakia. ^§^ refers to all countries of former Yugoslavia (*) because no locality details are available according to original data [65]. Records are given in Table 3.

Based on the previously published literature a total of 98 parasite species have been reported from the 14 resident *Myotis* species in Europe (Table 3). Most of the species were recorded in Germany, Italy, Poland and Slovakia (Figure 5). In some cases detailed specifications of location were missing. The list includes records of 42 parasitic arachnids, 30 insects, 13 nematodes, 8 digeneans and 4 cestodes. With about 43% (n=42) of the species, Arachnida is the most species rich class in the list of parasites. It is represented by the orders Mesostigmata (n=27), Ixodida (n=6), Sarcoptiformes (n=4), Prostigmata (n=3) and Astigmata (n=1). The second largest class is Insecta, with about ca. 37% (n=30). In Europe three orders parasitize *Myotis*: flies (Diptera) (Superfamily Hippoboscoidea) (n=14), fleas (Siphonaptera) (n=13) and bugs (Heteroptera) (n=3). The phylum Nematoda is represented by 13 species (ca. 13%). Amongst these is the class Secernentea with the orders Strongylida and Spirurida, represented respectively by four and three species. The other six species belong to the class Adenophorea, order Enoplida. The class Cestoda is represented by four species (ca. 4%) which all belong to the order Cyclophyllidea. Eight species (ca. 8%) belonging to the order Plagiorchiida of the class Trematoda parasitize *Myotis* in Europe. Our calculations show that 12 of the 14 *Myotis* species serve as hosts of at least 5 different parasite species. Compared to other *Myotis* species from Europe, the parasite fauna of *M. myotis* shows the highest diversity.

**Table 3:** **Records of metazoan parasites of** ***Myotis*** **from Europe**

**Host**	**Occurence parasite with host**	**Citation**
*Myotis alcathoe*	Arachnida: *Ixodes ariadnae* (HU) *Ixodes simplex* (SK) *Ixodes vespertilionis* (SK,RO) *Spinturnix mystacinus* (CH,SK) Insecta: *Basilia italica* (SK)	[30-33]
*Myotis aurascens*	Insecta: *Basilia mongolensis nudior* (GR)	[34]
*Myotis bechsteinii*	Arachnida: *Argas vespertilionis* (IT) *Ixodes ricinus* (PL) *Macronyssus diversipilis* (GB) *Spinturnix bechsteini* (CH,DE,FR,IT,PL,SK)	[24,30,32,35-42]
	Insecta: *Basilia nana* (DE,SK) *Cimex pipistrelli* (n.d.) *Ischnopsyllus hexactenus* (DE) *Ischnopsyllus octactenus* (DE) *Ischnopsyllus simplex* (n.d.) *Nycteribia kolenatii* (n.d.) Nematoda: *Aonchotheca eubursata* (HU) *Molinostrongylus alatus* (n.d.) *Molinostrongylus vespertilionis* (n.d.)	
*Myotis blythii*	Arachnida: *Acarus penetrans* (nomen dubia) (IT) *Argas vespertilionis* (SK) *Binuncus parenzani* (IT) *Eyndhovenia euryalis oudemansi* (n.d.) *Hirstionyssus albatus* (IT) *Hystrionyssus arcuatus* (IT) *Ixodes ariadnae* (HU) *Ixodes vespertilionis* (SK) *Macronyssus granulosus* (IT) *Macronyssus rhinolophi* (IT) *Microtrombidium italicum* (IT) *Spinturnix acuminata* (n.d.) *Spinturnix myoti* (CH,SK) *Spinturnix kolenatii* (n.d.) *Spinturnix vespertilionis* (IT)	[24,30,32-35,43-46]
	Insecta: *Basilia nana* (n.d.) *Ischnopsyllus intermedius* (n.d.) *Nycteribia latreillei* (GR) *Nycteribia vexata* (IT) *Nycteribia pedicularia* (IT) *Nycteribia schmidlii schmidlii* (IT) *Nycteridopsylla eusarca* (n.d.) *Penicillidia conspicua* (n.d.) *Penicillidia dufourii* (CY,GR,IT)	
	Nematoda: *Aonchotheca moraveci* (ES) *Litomosa ottavianii* (IT) *Molinostrongylus alatus* (BG) *Molinostrongylus vespertilionis* (n.d.) *Physaloptera brevivaginata* (ES) *Trichuroides chiropteri* (IT)	
*Myotis brandtii*	Arachnida: *Argas vespertilionis* (DE) *Macronyssus crosbyi* (LV) *Macronyssus ellipticus* (GB) *Spinturnix kolenatii* (n.d.) *Spinturnix myoti* (DE,LV) *Spinturnix mystacinus* (CH,DE,SK) *Steatonyssus cavus* (LV) *Steatonyssus periblepharus* (DE)	[14,17,30,32,37,38,46]
	Insecta: *Basilia* italica (n.d.) Cimex *pipistrelli* (n.d.) *Ischnopsyllus hexactenus* (DE,LV) *Ischnopsyllus octactenus* (DE) *Ischnopsyllus simplex/mysticus* (not clearly separable) (DE) *Ischnopsyllus simplex* (DE) *Myodopsylla trisellis* (LV) *Nycteribia kolenatii* (DE)	
*Myotis capaccinii*	Arachnida: *Hystrionyssus arcuatus* (IT) *Spinturnix psi* (FR) *Steatonyssus periblepharus* (DE,HU)	[30,32,34,35,47,48]
	Insecta: *Nycteribia kolenatii* (IT) *Nycteribia latreillei* (IT) *Nycteribia pedicularia* (CY,GR,IT) *Nycteribia schmidlii schmidlii* (GR,IT) *Penicillidia conspicua* (IT) *Penicillidia dufourii* (GR,IT) *Phtiridium biarticulatum* (IT) *Rhinolophopsylla unipectinata* (IT)	
	Digenea: *Lecithodendrium linstowi* (IT) *Lecithodendrium rotundum* (IT) *Plagiorchis vespertilionis* (IT)	
	Nematoda: *Aonchotheca moraveci* (ES)	
*Myotis dasycneme*	Arachnida: *Argas vespertilionis* (PL) *Macronyssus crosbyi* (LV) *Macronyssus corethroproctus* (PL) *Spinturnix acuminata* (n.d.) *Spinturnix andegavinus* (n.d.) *Spinturnix dasycnemi* (SK) *Spinturnix myoti* (LV,PL) *Spinturnix mystacinus* (n.d.) *Steatonyssus cavus* (LV) *Steatonyssus periblepharus* (PL)	[17,24,32,36,37,48-50]
	Insecta: *Basilia nana* (n.d.) *Cimex pipistrelli* (n.d.) *Ischnopsyllus hexactenus* (DE,LV) *Ischnopsyllus intermedius* (DE) *Ischnopsyllus simplex* (n.d.) *Ischnopsyllus variabilis* (DE) *Myodopsylla trisellis* (LV) *Nycteribia biarticulata* (LV) *Nycteribia kolenatii* (n.d.) *Nycteribia pedicularia* (LV) *Nycteridopsylla pentactena* (DE)	
	Digenea: *Plagiorchis mordovii* (PL)	
	Cestoda: *Vampirolepis balsaci* (PL) *Vampirolepis skrjabinariana* (PL)	
	Nematoda: *Capillaria italica* (HU) *Molinostrongylus alatus* (n.d.) *Molinostrongylus vespertilionis* (n.d.)	
*Myotis daubentonii*	Arachnida: *Alabidocarpus intercalatus* (GB) *Macronyssus crosbyi* (LV) *Macronyssus diversipilis* (GB) *Macronyssus ellipticus* (GB) *Macronyssus flavus* (CZ) *Notoedres myoticola* (GB) *Nycteridocoptes poppei* (GB) *Spinturnix acuminata* (n.d.) *Spinturnix andegavinus* (CH,CZ,DE,PL,SK) *Spinturnix helvetiae* (SK), *Spinturnix kolenatii* (n.d.) *Spinturnix myoti* (LV), *Steatonyssus cavus* (LV) *Steatonyssus spinosus* (n.d.)	[14,17,24,30,32,35,37-39,49,51-55]
	Insecta: *Basilia nana* (n.d.) *Basilia nattereri* (SK) *Cimex pipistrelli* (n.d.) *Ischnopsyllus intermedius* (n.d.) *Ischnopsyllus hexactenus* (DE,LV) *Ischnopsyllus mysticus* (DE) *Ischnopsyllus octactenus* (DE) *Ischnopsyllus simplex* (DE,SK) *Ischnopsyllus variabilis* (n.d.) *Nycteribia kolenatii* (DE,IT,SK) *Nycteribia latreillii latreillii* (IT) *Nycteribia pedicularia* (LV,IT) *Nycteribia schmidlii schmidlii* (IT) *Nycteribia vexata* (IT) *Penicillidia monoceros* (DE) *Phthiridium biarcticulatum* (n.d.)	
	Digenea: *Lecithodendrium linstowi* (BY) *Plagiorchis vespertilionis* (BY,IT) *Prosthodendrium chilostomum* (BY) *Prosthodendrium longiforme* (BY) Nematoda: *Aonchotheca eubursata* (HU) *Capillaria romana* (BY) *Capillaria speciosa* (IT) *Molinostrongylus alatus* (n.d.), *Molinostrongylus spasskii* (n.d.), *Molinostrongylus tipula* (n.d.) *Molinostrongylus vespertilionis* (n.d.)	
*Myotis emarginatus*	Arachnida: *Eyndhovenia euryalis* (FR,PL) *Macronyssus rhinolophi* (n.d.) *Steatonyssus periblepharus* (DE), *Spinturnix emarginata* (ES,PL,SK)	[30,32,35,49,51,56,57]
	Insecta: *Basilia nana* (n.d.), *Basilia italica* (n.d.) *Cimex pipistrelli* (n.d.) *Ischnopsyllus emarginatus* (n.d.) *Ischnopsyllus simplex* (n.d.) *Penicillidia dufourii* (IT) *Phthiridium biarcticulatum* (n.d.) *Rhinolophopsylla unipectinata* (n.d.)	
	Digenea: *Lecithodendrium linstowi* (AT) *Prosthodendrium aelleni* (AT) *Prosthodendrium chilostomum* (AT)	
	Cestoda: *Myotolepis grisea* (AT)	
*Myotis myotis*	Arachnida: *Acarus penetrans* (nomen dubia) (IT) *Argas transgariepinus* (IT) *Argas vespertilionis* (DE) *Ichoronyssus scutatus* (SK, o.r./n.a.r.) *Ixodes ricinus* (DE,PL,o.r.) *Ixodes simplex* (DE,IT,PL) *Ixodes trianguliceps* (PL) *Ixodes vespertilionis* (IT,RO,SK) *Macronyssus cyclaspis* (n.d.) *Macronyssus diversipilis* (n.d.) *Macronyssus ellipticus* (GB) *Macronyssus flavus* (CZ) *Macronyssus granulosus* (IT) *Macronyssus rhinolophi* (IT) *Microtrombidium italicum* (IT) *Nycteridocoptes poppei*. (DE) *Nycteriglyphus tuerkorum* (?) (CZ) *Radfordia sicula* (IT) *Spinturnix acuminata* (n.d.) *Spinturnix myoti* (CH,DE,IT,PL,SK,o.r.) *Spinturnix mystacina* (n.d.) *Spinturnix psi* (IT) *Spinturnix vespertilionis* (IT) *Steatonyssus periblepharus* (IT,o.r.) *Steatonyssus spinosus* (DE,PL,SK) *Trombicula* sp. (DE)	[12-14,24,30,32,37-39,41,45,49,51,54-59]
	Insecta: *Basilia nana* (SK) *Basilia nattereri* (n.d.) *Cimex dissimilis* (DE,o.r.) *Cimex lectularius* (DE) *Cimex pipistrelli* (SK) *Ischnopsyllus elongatus* (DE) *Ischnopsyllus hexactenus* (DE) *Ischnopsyllus intermedius* (DE,IT) *Ischnopsyllus octactenus* (DE,o.r.) *Ischnopsyllus simplex* (DE) *Ischnopsyllus variabilis* (DE) *Nycteribia kolenatii* (DE,IT) *Nycteribia latreillei* (DE,IT,SK) *Nycteribia pedicularia* (IT) *Nycteribia schmidlii schmidlii* (IT) *Nycteribia vexata* (DE,IT,SK) *Nycteridopsylla eusarca* (DE,IT) *Nycteridopsylla longiceps* (DE) *Nycteridopsylla pentactena* (DE) *Penicillidia dufourii* (DE,IT,SK)	
	*Phthiridium biarcticulatum* (IT) *Rhinolophopsylla unipectinata* (n.d.)	
	Digenea: *Lecithodendrium linstowi* (AT) *Plagiorchis vespertilionis* (AT) *Prosthodendrium chilostomum* (AT)	
	Cestoda: *Cycloskrjabinia taborensis* (IT) *Vampirolepis balsaci* (AT,HU, n.a.r./o.r.) *Myotolepis grisea* (AT,HU) *Vampirolepis acuta* (HU)	
	Nematoda: *Aonchotheca moraveci* (ES) *Litomosa desportesi* (HU) *Litomosa ottavianii* (IT) *Molinostrongylus alatus* (*BA,BG,CH,*HR,*ME,*MK,*RS,*SI,o.r./n.a.r.) *Molinostrongylus spasskii* (n.d.) *Molinostrongylus vespertilionis* (n.d.) *Trichuroides chiropteri* (IT)	
*Myotis mystacinus*	Arachnida: *Argas vespertilionis* (DE,PL) *Ixodes* sp. (LV) *Ixodes vespertilionis* (SK) *Macronyssus ellipticus* (GB) *Macronyssus flavus* (DE) *Spinturnix kolenatii* (n.d.) *Spinturnix myoti* (LV,SK) *Spinturnix mystacinus* (DE, CH,PL) *Steatonyssus periblepharus* (DE,GB) *Steatonyssus spinosus* (n.d.)	[14,17,24,30,32,35,39,40,43,47,49-51,55,56]
	Insecta: *Basilia nana* (n.d.) *Basilia nattereri* (n.d.) *Basilia italica* (IT,SK) *Cimex pipistrelli* (n.d.) *Ischnopsyllus hexactenus* (DE,LV) *Ischnopsyllus mysticus* (DE) *Ischnopsyllus octactenus* (DE) *Ischnopsyllus simplex* (DE,SK) *Ischnopsyllus variabilis* (n.d.) *Myodopsylla trisellis* (LV) *Nycteribia kolenatii* (n.d.) *Nycteribia pedicularia* (IT) *Nycteridopsylla longiceps* (DE) *Nycteridopsylla pentactena* (DE) Digenea: *Lecithodendrium linstowi* (AT), *Plagiorchis vespertilionis* (IT) *Prosthodendrium chilostomum* (AT)	
	Nematoda: *Molinostrongylus alatus* (n.d.) *Molinostrongylus spasskii* (n.d.) *Molinostrongylus vespertilionis* (n.d.)	
	Cestoda: *Vampirolepis balsaci* (PL)	
*Myotis nattereri*	Arachnida: *Argas vespertilionis* (DE) *Ixodes vespertilionis* (DE) *Macronyssus diversipilis* (GB) *Macronyssus ellipticus* (GB) *Macronyssus flavus* (DE) *Spinturnix andegavinus* (n.d.) *Spinturnix myoti* (DE,FR,SK) *Spinturnix mystacinus* (n.d.) *Spinturnix psi* (n.d.)	[14,24,30,32-38,45,46,49,55,57,60]
	Insecta: *Basilia nattereri* (SK) *Ischnopsyllus hexactenus* (DE) *Ischnopsyllus intermedius* (DE) *Ischnopsyllus octactenus* (DE) *Ischnopsyllus simplex* (DE,SK) *Ischnopsyllus variabilis* (n.d.) *Nycteribia kolenatii* (n.d.) *Nycteribia latreillei* (n.d.) *Penicillidia dufourii* (CY)	
	Digenea: *Allassogonoporus amphoraeformis* (BY) *Mesotretes peregrinus* (ES) *Plagiorchis koreanus* (IT) *Plagiorchis vespertilionis* (IT) *Prosthodendrium aelleni* (AT)	
	Cestoda: *Myotolepis grisea* (PL) *Vampirolepis acuta* (HU)	
	Nematoda: *Aonchotheca eubursata* (HU) *Capillaria italica* (HU,IT) *Molinostrongylus alatus* (n.d.)	
*Myotis oxygnathus	Arachnida: *Ichoronyssus jacksoni* (AT) *Ichoronyssus scutatus* (n.d.) *Macronyssus rhinolophi* (n.d.) *Steatonyssus spinosus* (n.d.)	[36,47,48,57,61-63]
	Nematoda: *Litomosa desportesi* (HU) *Physaloptera brevivaginata* (HU)	
	Cestoda*: Myotolepis grisea* (HU)	
*Myotis punicus*	Arachnida: *Spinturnix myoti* (IT,FR)	[64]

Given are the parasite-records of 14 European *Myotis* species. If available, country of record is mentioned. Abbreviations: *AT* Austria, *BA Bosnia and Herzegowina, *BG* Bulgaria, *BY* Belarus, *CH* Switzerland, *CY* Cyprus, *CZ* Czech Republic, *DE* Germany, *ES* Spain, *FR* France, *GB* Great Britain, *GR* Greece, *HR Croatia, *HU* Hungary, *IT* Italy, *LV* Latvia, *ME Montenegro, *MK The former Yugoslav republic of Macedonia, *PL* Poland, *RO* Romania, *RS Serbia, *SI Slovenia, *SK* Slovakia, *n.d.* no data given. *n.a.r.* new area record, *o.r.* own record. *All countries of former Yugoslavia were included because no locality details are available. *may not distinct on species level, treated as subspecies or synonym of *M. blythii* by some authors [62,63].

## Discussion

### Field study

#### Condition of juveniles and virus detection

The specimens investigated were not weaned off juveniles born in 2013. The fur of the specimens was fully developed; however, they had never been out of the roosting habitat. This was indicated by the short forearm length with less than 58 mm, which is considered the minimum length necessary for flight according to Kulzer [66]. The high parasitic infections of juveniles found here is a rather common phenomenon in bats. Juvenile *M. myotis* are more frequently infected than adults, most likely due to the fact that the immune system of juvenile bats is hardly developed [67]. Grooming, considered one of the most effective mechanisms of ectoparasite reduction, is also ineffective at a low age [68], thus, the harm for juvenile specimens is expected to be higher than in adult specimens. However, the juvenile *M. myotis* sampled here seem to have compensated for the harm caused by the parasites because of the good nutritional condition. No correlation between parasite burden and condition of host was found (Figure 3 and 4). Our assumption is supported by experimentally quantified results as described further below. The results of testing for viruses yielded no indication of a *Lyssavirus* specific antigen. However, as the infection rates of European *M. myotis* populations vary according to the type of *Lyssavirus* involved as well as between regions and through time [3,10], infection rates in Germany might still be worth investigating.

#### Parasite parameters

Sampled *M. myotis* were heavily parasitized (Table 2). The intensities and therewith indices of relative frequency (pi %) varied strongly within the eudominant [69] species *I. scutatus*, *S. periblepharus*, *S. myoti*, *C. dissimilis* and *V. balsaci. S. periblepharus* was the most dominant species with up to 1,288 specimens on a single host, which is the highest amount of this parasite species found in bats so far. In addition, we estimate that the number of mobile ectoparasites in living *M. myotis* is higher because ectoparasites leave the host after death. *I. octactenus* and *C. dissimilis* are very mobile and we estimate the highest differences of parasite burden compared to living bats within these species. *I. ricinus*, *I. scutatus*, and *S. periblepharus* are less mobile, *S. myoti* is hardly mobile because of the high grade of specialisation. We estimate that the infection rates differ slightly within the first three species and hardly differ in *S. myoti*. Therefore the results are nearly transferable to living *M. myotis* within this species. Despite the extreme infection rates found in one individual, the parasite fauna and intensities found here coincide with a regular parasite pattern within a *M. myotis* population [16]. Therefore, especially juvenile *M. myotis* form the habitat for a wide range of different associated species and are an integral part of the ecosystem. A sex-specific difference in the degree of parasitism in juvenile *M. myotis* was absent which confirmed earlier findings by Christe et al. [68]. Bigger parasites are likely to remove more energy from the host. Hence, the index of relative frequency and parasite size must be taken into account to assess damage to the host. Giorgi et al. [70] postulated a rise in overall metabolism rate of *M. myotis* of approx. 0.5% with each additional *Spinturnix myoti* individual. However, in contrast to the lab-based study by Giorgi et al. [71], a correlation between mite infections and index of condition in free-living *M. myotis* bats was not observed in this study (Figures 3 and 4).

#### Ectoparasites

Generally, the high infection rates with ectoparasitic mites can be explained by the typical phenological process of parasitism in *M. myotis* [16]. The infection rates found here during July depict probably the highest infection rates that can be expected during the year. *Spinturnix* mites are strictly Microchiroptera specific parasites [67]. *S. myoti*, the most common *Spinturnix* species found, uses *M. myotis* as the main host, but it also accepts other *Myotis* species and genera such as *Pipistrellus*, *Plecotus* and *Vespertilio* [14,17]. The prevalence found here is comparable to a study on juvenile *M. myotis* populations from Germany and Portugal where slightly lower Spinturnicidae (without further determination) prevalence of 94.7% and 96.6% were found [16]. Christe et al. [71] were also able to record comparable prevalence of 99.5% from *S. myoti* in juvenile *M. myoti*s from Switzerland. High prevalence of *Spinturnix* mites are a result of their extreme specialisation and adaptation to bats as hosts. *Spinturnix* females possess an evolutionary advantage compared to other parasites, in that they bear deuteronymphs with shorter development on hosts rather than laying eggs [67]. Contrastingly, parasitism of the two rare species (in the sense of Zander [69]) *Ischnopsyllus octactenus* and *Ixodes ricinus* were under represented in the bat population.

#### Endoparasites

Regarding the species composition of endo-and ectoparasites, our results vary widely from other observations, e.g. from Slovakia or other populations in Germany [12,16]. Most of the former recorded parasite species belong to completely different systematic taxa (Table 3). Based on our own practical work as well as the information based on other literature (see section further below on parasites of European *Myotis* populations), *M. myotis* serves as host for a wide range of different parasite species. Within the group of endoparasites, *Molinostrongylus alatus* and *Vampirolepis balsaci* are of special interest. Both have not previously been recorded on *M. myotis* in Germany. Only little is known about their life cycle. Single host life cycles as well as vertical transfers are described for numerous nematodes, which probably also applies to *M. alatus*. The infestation rates of *M. alatus* in the sample was clearly lower (I=1) than in the few cases reported in the literature, e.g. a prevalence of 65.21% and intensity of 1–28 with *M. myotis* as a host, and a prevalence of 46.66% and intensity of 1–45 in the closely related *M. blythii* [24]. Even more surprising than the infestation of juvenile *M. myotis* with *M. alatus* is the occurrence of *V. balsaci.* Based on life cycles of other cestodes following strategies of transmission are possible: As in nearly all Cyclophyllidea, a life cycle involving at least two hosts can be assumed for *V. balsaci*. Intermediate hosts involved are most likely fleas, for example of the genera *Ischnopsyllus* and *Nycteridopsylla*, which are frequent parasites of *M. myotis. Ischnopsyllus octactenus* was found in the studied population from Hesse. As adult fleas are exclusively haematophagous, this mode of bat infection would necessarily entail an infection of larval fleas by ingesting eggs containing infectious larvae of *V. balsaci*. After metamorphosis into adult fleas an oral uptake of fleas by the bats would follow and complete the cestode life-cycle. Because the cestode and the possible intermediate host (*I. octactenus*) were found in the bat population, this way of transmission seems to be very likely. A similar infection pathway has been described for the double-pore tapeworm (*Dipylidium caninum*) [72]. Apart from fleas, mites might be intermediate hosts for *V. balsaci* and be ingested during grooming with the plants that are fed on as described for other vertebrate cestodes [73]. However, as the studied *M. myotis* were still not weaned off and had probably never left the breeding burrow or showed grooming behaviour, this mode of infection does not explain the infections with *V. balsaci*. A further possibility is auto-infection through the oral uptake of embryonated eggs in the faeces. A similar life cycle of autoinfection with a single host has already been described in the related species *Hymenolepis nana* (syn. *Vampirolepis*) [74]. It is striking that the prevalence of *M. alatus* (P=3.3%) and *V. balsaci* (P=70%) are extremely different. One explanation could be the different modes of infection, with *V. balsaci* infections probably being more time-consuming than the supposed direct infection with *M. alatus*. Beside the two endoparasite species, *I. scutatus* and *S. periblepharus* (Arachnida, mites) are the four species with a new distribution record on *M. myotis* within Germany. Our findings of the four new parasite species that use *M. myotis* as a host show that new insights into the *Myotis* parasites are still common and more records can likely be expected.

### Parasites of *Myotis* in Europe

#### European parasite fauna

To obtain an overview of the parasite fauna of *Myotis* species in Germany and other European countries, available published records of metazoan parasites of all European *Myotis* species were collated. The eight recorded parasite species that were found in the *M. myotis* population at the site in Gladenbach have already been noted to be parasites of *M. myotis* and of other *Myotis* species in Europe and are contributing about 25% of the parasite species on *Myotis* in Germany (Figure 5).

**Figure 6:**
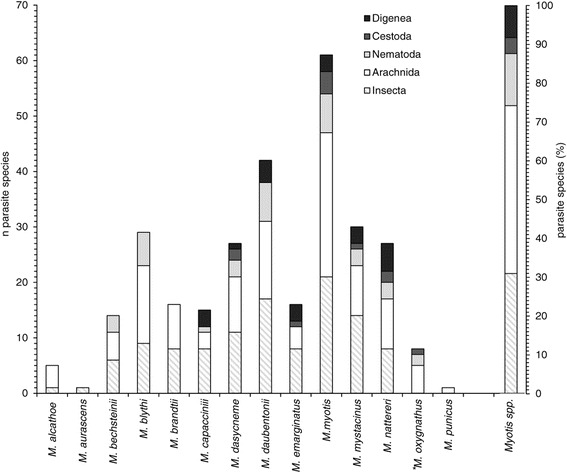
**Host specification of European** ***Myotis*** **parasites.** Shown are the total numbers and composition of the parasite species of each European *Myotis* species. The parasite species are combined in the five taxonomic groups. Furthermore, the percentages of the five taxonomic groups of all European *Myotis* parasites are shown. *Myotis* species are listed in alphabetical order. * may not distinct on species level, treated as subspecies or synonym of *M. blythii* by some authors [62,63].

More than half of the species of the European parasite fauna of *Myotis*, currently 98 species, belong to a few, bat-specific genera of different classes. Arachnida of the order Mesostigmata, and within these the genera *Macronyssus* and *Spinturnix* are dominating the parasite fauna and are contributing approx. 21% to the parasite fauna of *Myotis*. Both genera occur as parasites of bats worldwide and sometimes reach an extreme degree of specialization on their hosts, e.g. with co-differentiation of *Myotis punicus* and its parasite *Spinturnix myoti* within different geographic lineages [64]. New species are regularly described in both genera, whereby the taxonomy has been affected by numerous changes [26,27]. The strictly bat specific Diptera and rather heteroxenous Siphonaptera are numerically also strongly represented among the European parasite fauna of *Myotis*. Similarly, most species of Cestoda belong to the bat-specific genus *Vampirolepis*. Based on our compilation we can state that the largest part of the digeneans, nematodes, arachnids, and insects was found only on a single host. With rising host numbers the number of parasite species parasitizing the same host decreases. Within the cestodes only one different species could be observed parasitizing the same host. The insect and arachnid species are the most diverse group within the European *Myotis* parasite fauna (Figure 6). The highest number of parasites is reached by *M. myotis*; we assume this coincides with its great distribution pattern.

#### Distribution pattern and composition of parasite species in Myotis

The distribution pattern of bat parasites could be explained by the effects of the geographical barriers affecting bat distribution. The West Siberian Plain in the East forms a natural barrier between the European-Ural and Siberian-Far Eastern complexes [75]. In the South, the Strait of Gibraltar functions as a natural barrier between Europe and Africa [6]. Geographical isolation within Europe is supposed to have led to species diversification within the few classes or genera of bat parasites that became established. Furthermore, the close relationship of parasite and host based on co-speciation at molecular level allows for the reconstruction of former distribution patterns of the host species [64]. Another factor that relates parasites to their host species is the mode of life of bats, i.e. their ability to fly reduces the likelihood of an infection with unspecific parasite species. For example infections with unspecific *Ixodes* (ticks) in bats are rather uncommon [43]. Similarly, only two bat-specific families, Streblidae and Nycteribiidae (Diptera, superfamily Hippoboscoidea), constitute more than 68% of the bat parasites of South and Central America [76]. Additionally, the relatively high evolutionary age of bats compared to other mammals has probably played a role for the specific recent bat parasite composition. Earliest records date back to the early Eocene, but ancestors of modern bats might have already existed in the Paleocene as early fossil bats display the morphological characters of more recent bats [77]. Furthermore, Simmons [78] considered it likely that all recent bat families already existed by the late Eocene. This view is supported by the fact that remains of the oldest fossil of the bat-parasitic Streblidae are at least 15 million years old [79]. It seems reasonable to assume that not only ecological requirements and the way of life of early bats were similar to modern ones, but that also a specialized parasite fauna existed very early, worldwide and also in Europe. It was shown that the European wide composition of the *Myotis* parasite fauna is dominated by a few specific taxonomic groups in Europe. Based on these findings we propose that in the worldwide fauna of bat parasites a correspondingly long specialization on hosts might have similarly led to the dominance of particular taxonomic groups. This co-speciation led to the high diversity of species associated with bats. More studies covering a larger range are needed to clarify the today status of bat parasite species worldwide. Results would provide new insights into the parasite-host co-evolution processes and help to better understand the ecology of bats.

## Conclusions

The juvenile greater mouse-eared bats from the collecting point in Hesse (Germany) showed high parasite load and diversity. Due to the low age of the specimens, infection rates were high. Among the eight parasite species found, *I. scutatus* and *S. periblepharus* (Arachnida, mites), *V. balsaci* (Cestoda) and *M. alatus* (Nematoda) were recorded for the first time in Germany. The findings state that especially juvenile *M. myotis* serve as a habitat for a great range of parasite species. Therewith, they form an integral part of the ecosystem and contribute to species diversity in high amount. Especially the finding of endoparasitic cyclophyllidean cestodes that have usually at least a two-host lifecycle is, considering the stationary behaviour of the juvenile bats, rather unusual and suggests a non-predatory transmission mechanism (e.g. described for *Hymenolepis nana*). We assume that the number of parasites in juvenile *Myotis* is generally higher than in adults due to the only later acquired immune competence and behavioural adaptations. A complete overview of the European *Myotis* parasite fauna was given and revealed that a few parasite taxa dominate the recent European *Myotis* parasite fauna. Thus, a parasite-host co-specification in this unique taxonomic group is suggested.
